# Is our immune system a powerful vaccine factory?

**DOI:** 10.1590/1678-4685-GMB-2020-0468

**Published:** 2021-08-06

**Authors:** Michel E. Beleza Yamagishi

**Affiliations:** 1 Embrapa Informática Agropecuária, Campinas, SP, Brazil.

The human immune system is supposed to defend our organism from disease-causing agents such as viruses and bacteria. It performs this task using different strategies from directly killing the pathogens to reducing their viability (Nicholson, 2016). For instance, it is known that one member of the double-domain cytidine deaminase APOBEC, namely APOBEC3G, inhibits a broad range of retroviruses, such as HIV, by hypermutating their sequences (Mangeat *et al*., 2003). The basic idea is to produce a premature stop-codon or modify some important protein to the virus propagation cycle. There are at least two gene families that edit RNA, DNA or both (Navaratnam and Sarwar, 2006; Avesson and Barry, 2014; Chemudupati *et al*., 2019), which suggests that the virus sequence hypermutation (VSH) mechanism may be an evolutionary defense tool (Vieira and Soares, 2013).

However, to the best of our knowledge, the scientific community has failed to realize that the VSH mechanism may be an important player in the co-evolution of viruses and hosts (Kerr *et al*., 2017), producing attenuated virus strains that eventually become dominant. For instance, some mutations may impair the viral pathogenicity. Nothing prevents these mutated viruses to leave the host and, because infected people do not get badly ill to propagate faster than the wild type. In a real-life situation, these attenuated viruses could act just like a vaccine, conferring immunity to the population. Perhaps, the VSH mechanism evolutionary function is to generate, within each infected cell, i.e., in a massive parallel production, mutated strains that could train the immune system against the virulent wild type. If this conjecture proves correct, then the immune system has a two-fold mission: *to safeguard our organism by destroying the threatening agents and to protect our species, acting like a powerful vaccine factory*. But how realistic is this speculation?

Since its very beginning in Wuhan, the SARS-CoV-2 virus has been sequenced hundreds of thousands of times, and new sequences are still being deposited every day. Thousands of single nucleotide polymorphisms, called SNPs, had been reported, and those SNPs helped to trace back the virus’ geographic origin and its probable starting date (van Dorp *et al*., 2020a). Of course, the very existence of SNPs is consistent with the viruses “quasispecies” theory, which states that high mutation rates are expected to occur just by chance (Crotty *et al*., 2001). Yet, *those mutations could also be a sign of the action of the VSH mechanism*. Despite their different etiology, both processes can co-exist: the virus evolution *versus* the VSH mechanism. 

Is there any evidence that the VSH mechanism is mutating the SARS-CoV-2 virus? The first evidence that a host-dependent RNA editing mechanism was mutating the SARS-CoV-2 sequence appeared as soon as March 3^rd^ 2020. Dr. Conticello’s group identified signatures of RNA editing, namely, Adenosine-to-Uracil changes from ADAR deaminases and Cytosine-to-Uracil changes from APOBEC ones (Di Giorgio *et al*., 2020). This amazing finding was independently confirmed by Dr. Balloux’s group which analyzed 46,723 SARS-CoV-2 genomes isolated from patients worldwide, in their own words: “*we observed an enrichment in CCA and TCT 3-mers containing a variable base in their central position, which are known targets for the human APOBEC RNA-editing enzyme family*” (van Dorp *et al*., 2020b). The authors of those two manuscripts did not draw the straightforward and bold conclusion of their own work. It was Dr. Simmonds (Simmonds, 2020) who dared to uphold that: “*the finding that a large proportion of sequence change in SARS-CoV-2 in the initial months of the pandemic comprised C→U mutations in a host APOBEC-like context provides evidence for a potent host-driven antiviral editing mechanism against coronaviruses more often associated with antiretroviral defense.*” This last statement extends the VSH mechanism beyond retroviruses, but it fails to acknowledge that it may be an important player in the co-evolution of viruses and hosts.

There are three main objections that should be addressed. The first is that most of the immune system induced mutations are silent, i.e., they preserve protein sequences (van Dorp *et al*., 2020a), and, unfortunately, the scientific community is looking for non-silent mutations that could produce either more virulent or attenuated strains. However, silent mutations have at least two advantages over non-silent ones from an immunological point of view: (1) they, by definition, preserve the protein sequence which could be used to train the immune system against the wild type, and (2) they may “*decrease translational load on the host per unit of expression*” (Cheng *et al*., 2020) which could, at least in principle, reduce SARS-CoV-2 virulence.

The second concern is that the SARS-CoV-2 virus has not changed significantly. Despite thousands of mutations having been reported so far, the average pairwise difference (APD) between any two SARS-CoV-2 genomes is 9.6 SNPs (van Dorp *et al*., 2020a). Considering that the SARS-CoV-2 reference genome (NC_045512.2) has 29,903 nucleotides, the APD is less than 0.033%, which confirms that the SARS-Cov-2 virus is almost unchanged. Nevertheless, the question that begs to be answered is: how many silent mutations are necessary to affect protein expression or function? Surprisingly enough, the answer to this important question is *just one* mutation (Kimchi-Sarfaty *et al*., 2007). There are also several papers discussing how important a few silent mutations can be to protein expression and function (Komar, 2007; Harrigan *et al*., 2008; Plotkin and Kudla, 2011; Sauna and Kimchi-Sarfaty, 2011). Therefore, silent mutations induced by the immune system should at least be taken seriously. 

Maybe, a subset of those silent mutations induced by the immune system may produce an attenuated SARS-CoV-2 strain, which is the last issue I will address. Recently, Dr. Ozer’s group has reported a clade of SARS-CoV-2 associated with lower viral loads in patients (Lorenzo-Redondo *et al*., 2020). As expected, the authors emphasize the role of a non-silent mutation, namely D614G, within the spike protein. However, they also reported two silent mutations *C→U.* The first occurred within the Nsp14 protein (orf1ab, position 18060) and helped to distinguish the Clade 2, supposedly with lower viral load, from Clade 3. The second was within the Nsp3 protein (Orf1a, position 3037) and helped to sort out Clade 2 from Clade 1. As the authors did not determine causal relationships between mutations and phenotype, it is possible that those silent mutations may play an important role as well; otherwise, Clade 3 should have presented a lower viral load because it also shared the same non silent spike mutation.

This letter intends to call attention to the question whether the immune system is also a powerful vaccine factory. It goes without saying that the “vaccine factory” wording is just a metaphor; it does not imply the existence of a targeted goal. There is already sufficient evidence that our immune system is actively mutating the SARS-CoV-2 sequence. No one fully understands the impact to the virus cycle of those mutations, particularly the silent ones. It is necessary to probe and study the relationship between those immune system induced mutations and the viral cycle. Our technology is sufficiently advanced to address the problem. We are just missing to put together a multidisciplinary research group to work on it. The paradigm of vaccine production (Plotkin, 2009) needs to evolve to minimize the time taken and to reduce the costs. Throughout the centuries, men have always looked at nature for inspiration. If the VSH mechanism creates natural vaccines, then what can we learn from it? Are the silent mutations the best way to produce attenuated viruses? Is it possible to emulate it ‘*in vitro*’? Can we use the virus' own capsid to deliver the vaccines?
